# Concurrent Occurrence of Lobular Capillary Haemangioma and Port-Wine Stain: A Case Report and Literature Review

**DOI:** 10.7759/cureus.38642

**Published:** 2023-05-06

**Authors:** Sasti Priya, Karthik Rajaram Mohan, Ravikumar Pethagounder Thangavelu, Saramma Mathew Fenn, Kumar Appusamy

**Affiliations:** 1 Oral Medicine and Radiology, Vinayaka Mission’s Sankarachariyar Dental College, Vinayaka Mission’s Research Foundation-Deemed University, Salem, IND

**Keywords:** hmme-pdt therapy, port-wine stain, pulsed-dye lasers, hamartoma, capillary haemangioma

## Abstract

A port-wine stain is a type of non-neoplastic hamartomatous malformation of capillary blood vessels, resulting from ectatic capillaries present from birth. Lobular capillary hemangioma is a form of capillary hemangioma that occurs from hamartomatous malformation of capillaries. In our report, we discuss the rare case of both port-wine stain and capillary haemangioma on the gingiva in a 22- year-old young male.

## Introduction

A port-wine stain is a hamartomatous malformation of the capillary blood vessels. Such lesions are present from birth but become more apparent after the pubertal age [1]. Port-wine stains do not undergo regressive changes, unlike other haemangiomas such as salmon patches, which undergo involution or regressive changes by the formation of fibrous scar or fine telangiectasia on the surface of the skin where they occur. Some persist as nodular growth due to the presence of ectatic blood vessels in them [2]. Dysregulation of vascular mitogen-activated protein kinase (MAPK) and/or phosphoinositide 3-kinase (PI3K) signalling during human embryonic development, as well as somatic mutations of *GNAQ* and *P13K*, play a role in the pathogenesis and progression of Port Wine Stain [1].

The term "lobular capillary haemangioma" represents a form of hamartomatous malformation affecting the capillary blood vessels. Lobular capillary haemangioma occurs as bright red, raised nodular growth on the gingiva, and has an increased tendency to bleed [3]. Lobular capillary haemangioma occurs concurrently in patients with port-wine stain. It can occur in Sturge-Weber syndrome, in which patients have a history of seizures and a risk of increased intraocular pressure (glaucoma). The cerebral cortex underlying the leptomeningeal (arachnoid and pia mater) vascular malformations usually become dystrophic, non-functional, and calcified producing a tram-track sign in the lateral cephalometric skull radiographs [4]. The concurrent occurrence in a patient of such a lobular capillary haemangioma with port-wine stain is discussed here.

## Case presentation

**Figure 1:**
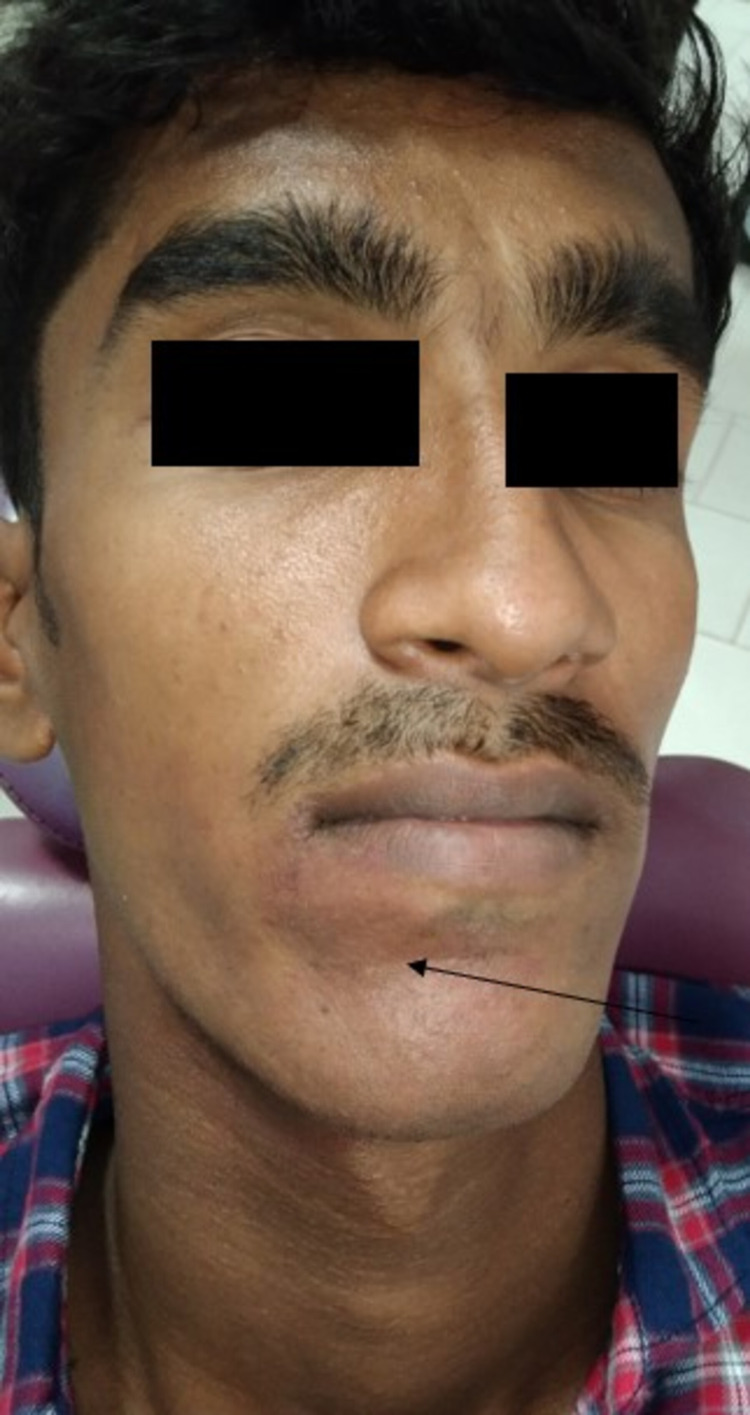
Extraoral examination revealed a port-wine stain below the right side vermilion border of the lower lip

A 22-year-old male reported with a chief complaint of growth on the gums, present from birth. On eliciting the history of presenting illness, it started as a small growth involving the gingiva and has gradually progressed to the present size. The growth became more prominent over the period of the past seven months. The growth interfered with speech and he had mild discomfort of bleeding, only while brushing his teeth. On extraoral examination, a patch with a pinkish hue was seen on the skin of the face near the chin and below the lower lip on the right side (Figure 1).

**Figure 2:**
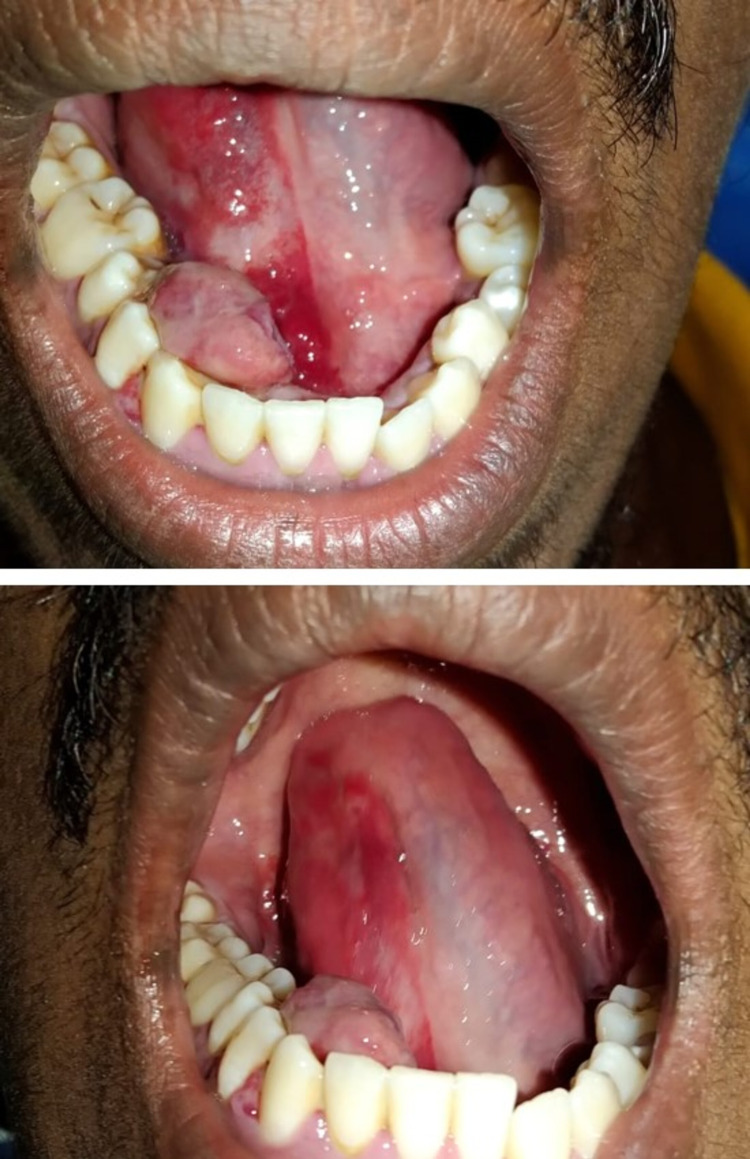
Intraoral clinical photograph revealed a growth with purplish hue on the gingiva on the lingual aspect in relation to 26, 27, 28, 29 regions and bright, fiery-red area only on the right side ventral surface and floor of the mouth not crossing the midline

History revealed he had no episodes of any seizures. General examination revealed his vitals are stable. On intraoral examination, a growth was seen on the lingual aspect of the gingiva in relation to the 26, 27, 28, 29 tooth regions. It measured approximately 1.5 x 2 cm in diameter approximately; the surface showed discrete areas of purplish hue. The right side of the floor of the mouth and the ventral surface of the tongue appeared bright red, not crossing the midline (Figure 2).

On palpation, the growth was firm in consistency, non-tender, sessile, non-pedunculated, compressible, and reducible, showing an increased tendency to bleed. The growth showed a positive modified diascopy test when performed with the back of the mouth mirror (Video 1).

**Video 1:** Modified diascopy test is positive for blanching

**Figure 3:**
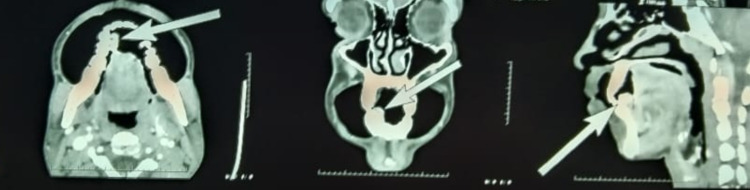
Contrast-enhanced CT (axial, coronal, and sagittal section) revealed a feeding artery on the gingiva on the lingual aspect in relation to 27, 28, 29 regions

The differential diagnosis for the growth on the gingiva includes fibroma, peripheral ossifying fibroma, pyogenic granuloma, peripheral giant cell granuloma. Fibroma is a universal tumour that can occur anywhere in the body. Usually, fibroma inside the oral cavity appears clinically as a growth showing the same colour as adjacent mucosa. Pyogenic granuloma usually occurs from local irritating factors such as supragingival and subgingival calculus deposits [5]. In our case, local factors such as supragingival calculus are not present, hence pyogenic granuloma is ruled out. Peripheral ossifying fibroma can also occur as a sessile mass involving the gingiva [5]. Since there is a bright reddish area involving only the right lateral half of the floor of the mouth in relation to the vicinity of the growth in the 28, 29 regions, a feeding artery is suspected of supplying the growth on the gingiva. Hence, a contrast-enhanced CT is advised. The arterial phase of contrast-enhanced CT axial, coronal, sagittal sections revealed an enhanced contrast suggestive of a feeding artery supplying the growth on the gingiva in relation to the 27, 28, 29 regions (Figure 3).

**Figure 4:**
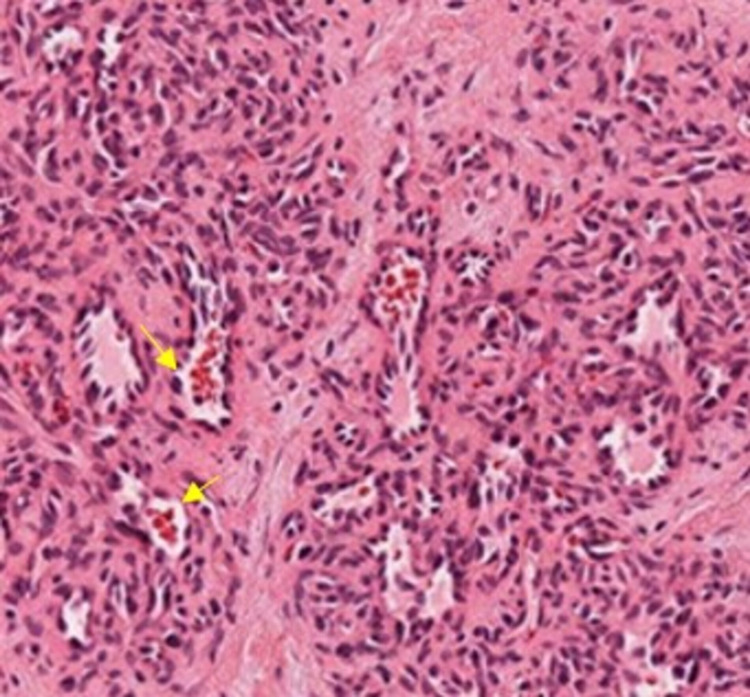
Histopathological photomicrograph revealed numerous capillaries, each lined by a single layer of endothelial cells (yellow arrows)

The growth on the gingiva is excised after presurgical precautions such as careful clamping of the major feeding vessels (lingual artery) supplying the growth on the gingiva. The compression was provided with the help of cotton gauze in the region for 15 minutes. The excised growth was sent for histopathological examination, which revealed numerous capillaries, each lined by a single layer of endothelial cells (Figure 4).

**Figure 5:**
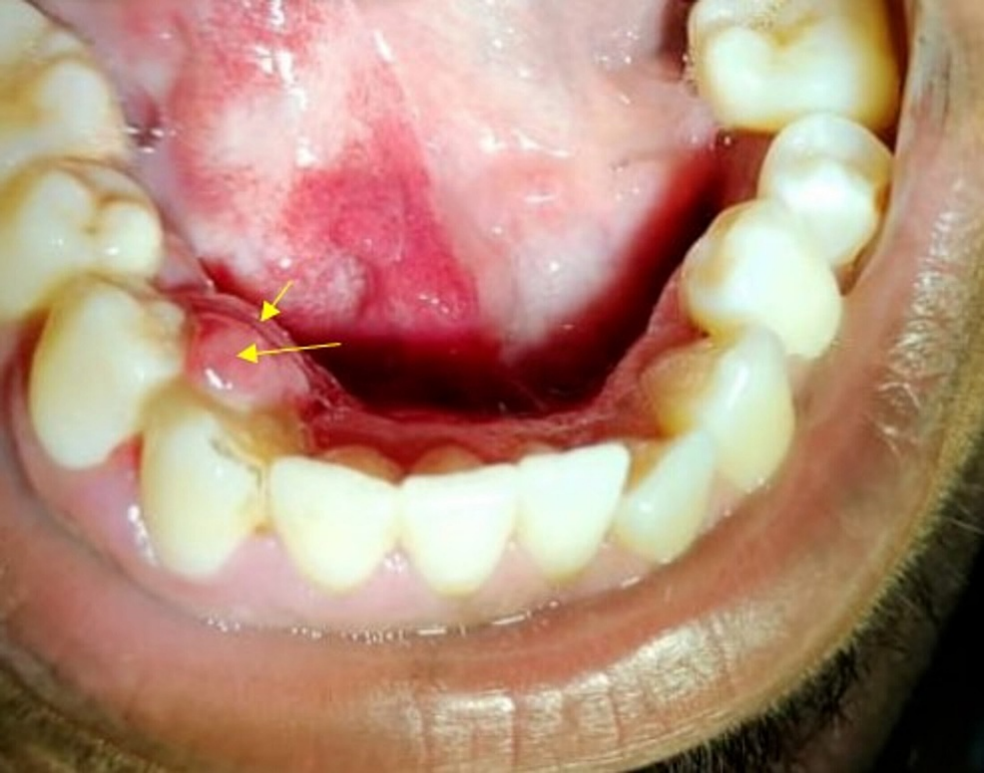
Postoperative intraoral clinical photograph in the one-month follow-up

The follow-up intraoral photograph after a month revealed a mild inflammatory swelling on the lingual aspect of marginal gingiva and interdental papilla in relation to 28, 29 region (Figure 5).

The other management options for capillary haemangioma include: Tixel-induced rapamycin delivery following pulsed dye laser, hematoporphyrin monomethyl ether photodynamic therapy (HMME-PDT), sirolimus drug therapy [6-8].

## Discussion

The port-wine stain is a non-neoplastic hamartomatous proliferation of capillaries resulting in nodular growth, as a result of ectatic blood vessels [2,9-12]. A port-wine stain clinically presents from birth and such lesions do not regress or undergo involution over a period of time. Port-wine stains are formed by somatic mutations of *GNAQ1*, *P13K* and dysregulation of vascular MAPK and/or PI3K signalling vascular pathway during human embryonic development [1].

Port-wine stain was first described as granuloma pyogenicum. It is a form of nevus flammeus affecting the capillaries in the dermis and is more common on the external surface of the facial skin. They are distributed along the dermatomal branches supplied by the trigeminal nerve. They occur clinically as asymptomatic, nodular growth involving the gingiva, non-tender, compressible, and reducible on palpation and also showing a positive modified diascopy test, on the application of pressure ( by the back end of the mouth mirror). Such growth was friable and tendency to bleed [6]. The unilateral distribution of the reddish colour not crossing the midline in relation to the right ventral surface of the tongue and floor of the mouth provided a clue to the diagnosis. Such encountered growth must be carefully dealt with and a proper clinical and radiographic examination should be done before a surgical excision is attempted [7,8,13-15]. The various investigations and management modalities for port-wine stain are listed in Table 1.

**Table 1:** Various Investigation and management modalities for port-wine stain

Investigation modalities	Management modalities
Dark ground illumination with confocal reflectance microscopy	Pulsed dye lasers (wavelength=585 nm)
Artifical RBCs - Haemoglobin vesicles	Cryotherapy: Cryoprobes with cryogen- liquid freeze-dried Nitrogen (-196^0^ C)
Dermatoscopy - capillaroscopy	Sclerotherapy: ethionamide
	Injection of antiangiogenic agents: sirolimus, retinoids, corticosteroids, propanolol
	Photosensitizers: artificial RBCs –haemoglobin vesicles in laser therapy
	Photodynamic therapy using hematoporphyrin (HMME-PDT)

The various research studies on the concurrent occurrence of lobular capillary hemangioma and port-wine stain are listed in Table 2. Swerlick et al. stated that the clinical appearance of nodular growth of lobular capillary hemangioma can be mistaken for Kaposi sarcoma, hence termed Pseudo-Kaposi sarcoma [16]. Sheehan et al. stated that the presence of pyogenic granuloma in areas of port-wine stain can mimick malignancy [17]. Rodins et al. reported the occurrence of pyogenic granuloma and port-wine stain in pregnant patients [18]. Madi et al. stated that port-wine stains are distributed along the branches of the trigeminal nerve [19]. Luna-Ceron et al. stated that lobular capillary hemangiomas are so called because they are characterized histologically by the lobular arrangement of capillaries in a fibrous stroma. They occur clinically as a bright red, exophytic, nodular growth on the gingiva, which are friable on slight provocation and are susceptible to bleeding. Port-wine stains also occur as bright red macules and characteristically do not cross the midline. Both lobular capillary haemangioma and port-wine stain have mutations in the G protein subunit alpha q (GNAQ), implying that lobular capillary haemangioma emerges from port-wine stain cells [20].

**Table 2:** Research studies on cases of concurrent occurance of lobular capillary haemangioma with port-wine stain

Author	year	Age/Gender	No. of cases
Swerlick et al. [16]	1983	22-year-old female, 8-year-old boy	2
Sheehan et al. [17]	2004	35-year-old male	1
Rodins et al. [18]	2011	23-year-old female	1
Madi et al [19]	2017	33-year-old female	1
Luna-Ceron et al. [20]	2021	21-year-old-male	1

## Conclusions

Haemangioma affecting the capillaries can occur as growth on the gingiva. such growth on the gingiva is friable easily and patients often complain of bleeding from the gingiva while brushing their teeth and may fear cancer and visit a dentist in doubt. Haemangiomas occurring in the gingiva are friable, have an increased tendency to bleed even on slight provocation during brushing. Bleeding, ulcer, airway compromise are some of the main complications that can be caused by a hemangioma if left untreated. Hence, it is always better to treat hemangioma occurring on the gingiva. Any growth involving the gingiva present from birth with a pinkish surface hue showing a positive modified diascopy test must be suspected for the vascular origin. Surgical procedures, which involve excision of such growth involving the gingiva, must be performed with precautions to avoid postoperative complications such as excessive bleeding, which can lead to hypovolemic shock.
